# Bipolar Disorder and Polysubstance Use Disorder: Sociodemographic and Clinical Correlates

**DOI:** 10.3389/fpsyt.2022.913965

**Published:** 2022-06-15

**Authors:** Andrea Aguglia, Antimo Natale, Laura Fusar-Poli, Andrea Amerio, Edoardo Bruno, Valeria Placenti, Eleonora Vai, Alessandra Costanza, Gianluca Serafini, Eugenio Aguglia, Mario Amore

**Affiliations:** ^1^Section of Psychiatry, Department of Neuroscience, Rehabilitation, Ophthalmology, Genetics, Maternal and Child Health, University of Genoa, Genoa, Italy; ^2^Istituto di Ricovero e Cura a Carattere Scientifico (IRCCS) Ospedale Policlinico San Martino, Genoa, Italy; ^3^Department of Clinical and Experimental Medicine, University of Catania, Catania, Italy; ^4^Department of Psychiatry, Faculty of Medicine, University of Geneva (UNIGE), Geneva, Switzerland; ^5^Faculty of Biomedical Sciences, Università della Svizzera Italiana (USI), Lugano, Switzerland

**Keywords:** bipolar disorder, substance use disorder, treatment, psychotic symptoms, polypharmacy

## Abstract

**Introduction:**

Patients with bipolar disorder (BD) often show comorbidity with substance use disorder (SUD) with a negative impact on clinical course, prognosis, and functioning. The role of polysubstance use disorder (polySUD) is understudied. The aim of the present paper is to evaluate the sociodemographic and clinical characteristics associated with BD and comorbid SUD, focusing on polySUD, in order to phenotype this specific group of patients and implement adequate treatment and prevention strategies.

**Methods:**

A cross-sectional study was conducted involving 556 patients with a primary diagnosis of BD (376 without SUD, 101 with SUD, and 79 with polySUD). A semi-structured interview was administered to collect sociodemographic variables, clinical characteristics, and pharmacological treatment. ANOVA and chi-square tests were used to compare the three groups. Significantly different variables were then inserted in multivariate logistic regression.

**Results:**

Patients affected by BD and polySUD were younger, and more frequently males and single, than patients with SUD or without SUD. Indeed, the prevalence of patients affected by BD and polySUD living in residential facilities was higher than in the other groups. Moreover, earlier age at onset, higher prevalence of psychotic and residual symptoms, involuntary hospitalization, and a family history of psychiatric disorders were associated with polySUD in patients suffering from BD. Lastly, patients with BD and polySUD were more likely to take four or more medications, particularly benzodiazepines and other drugs. At the multinomial regression, younger age, male gender, early age at onset, psychotic and residual symptoms, positive family history of psychiatric disorders, and use of benzodiazepines remained significantly associated with polySUD in patients with BD.

**Conclusion:**

Our findings show a specific profile of patients with BD and polySUD. It is important to conduct research on this topic in order to adopt specific therapeutic strategies, minimize the use of polypharmacy, and aim at full remission and mood stabilization.

## Introduction

Bipolar disorder (BD) is a severe, recurrent, and multifactorial psychiatric condition, characterized by the alternation of different phases, specifically hypomanic/manic and major depressive episodes (1). BD affects at least 2–3% of the general population and its onset typically occurs during adolescence or early adulthood (2), influenced often by seasonality and sunlight (3, 4). BD is often associated with several medical conditions, such as cardiovascular and endocrine-metabolic diseases (5–10) and higher inflammatory status (11, 12), leading to a worsening of the quality of life and functioning (13).

Substance use disorder (SUD), which is defined by the Diagnostic and Statistical Manual for Mental Disorders (DSM-5) criteria as “a cluster of cognitive, behavioral, and physiological symptoms indicating that the individual continues using the substance despite significant substance-related problems” (1), is a common issue in patients with BD. It has a negative impact on the illness course, including more difficulties to achieve intraepisodic remission and stabilization and maintaining adequate and full adherence to therapeutic strategies (14, 15).

The comorbidity between BD and SUD is frequent, as confirmed by several epidemiological studies. For example, the Epidemiologic Catchment Area (ECA) study found a higher prevalence of substance abuse in subjects diagnosed with BD type I (40.7%) than in the general population (16). In the National Epidemiologic Survey on Alcohol and Related Conditions (NERSAC) studies, patients with BD showed high rates of SUD (17–20), as well as in other epidemiological studies, with a prevalence ranging from 26 to 43% (21–23). In general, patients with BD tend to use illicit substances with a strong statistical association [odds ratio (OR): 4.96] with the following prevalence: alcohol (42%), cannabis (20%), and cocaine and amphetamine (17%) (24). However, the use of novel psychoactive substances has been also associated with young patients with BD more than controls and subjects with other psychiatric disorders (25, 26).

The reasons why patients with BD use illicit substances are different, including seeking relief by self-medication, improving psychic status, relieving tension and boredom, escaping from reality, achieving and/or maintaining elevated mood, and increasing energy (27). Furthermore, common genetic (i.e., polymorphism of the aldehyde hydrogenase and alcohol dehydrogenase, or 5HT_2C_ gene), socioeconomic, and environmental factors (i.e., access and availability of illicit drugs in the community, poverty), stressful events (i.e., trauma, physical, and sexual abuse), temperamental traits (i.e., a sensation of seeking behavior), lifetime suicide attempts, and psychiatric comorbidities (i.e., conduct disorder, cluster B personality disorder, and post-traumatic stress disorder) have been associated with SUD in patients with BD (24, 28, 29).

The comorbidity between BD and SUD is associated with several detrimental clinical characteristics, such as an earlier onset of illness, greater severity of the disorder, more rapid cycling with a higher risk of switching into (hypo)manic or mixed phases and frequent affective relapses, increased risk of suicide attempts or suicide, and accelerated progression of BD with higher prevalence of negative and cognitive symptomatology. Furthermore, patients affected by BD and SUD are characterized by treatment delay due to possible misdiagnosis, reduced response to lithium and other pharmacological treatments with worsening of adherence, presence of medical comorbidities (hepatitis C, acquired immune deficiency syndrome, and others), increased psychosocial problems, and poorer social support (27, 28, 30). Finally, illicit substances, particularly cannabis, are considered risk factors for the onset of BD (31, 32).

Although previous studies have recognized the negative impact of concurrent SUD in patients with BD, to the best of our knowledge, no studies have specifically focused on the factors associated with BD and polysubstance use disorder (polySUD). The present study aimed to evaluate the sociodemographic and clinical characteristics associated with BD and comorbid SUD, focusing specifically on polySUD. In fact, a detailed characterization of the phenotype of patients with BD and polySUD may help the identification and prevention of risk factors for multidrug abuse. This may in turn improve the clinical course and outcomes of BD and may help the building of a meaningful therapeutic relationship and the choice of the most effective treatments (28).

## Materials and Methods

### Sample and Assessment

A cross-sectional study was conducted involving 556 patients with a primary diagnosis of BD, consecutively admitted to the Section of Psychiatry, Department of Neuroscience, Rehabilitation, Ophthalmology, Genetics, Maternal and Child Health (DINOGMI), IRCCS Ospedale Policlinico San Martino, University of Genoa (Italy), during a period of 5 years (January 2017–December 2021).

The inclusion criteria consisted of the following: (a) inpatient status; (b) ongoing major depressive or (hypo)manic episode into a primary diagnosis of BD type I and II, according to the DSM-5 criteria (1); (c) 18 years of age or older; and (d) written informed consent to participate in the study.

The exclusion criteria were as follows: (a) primary diagnosis of schizophrenia and related disorders; (b) pregnancy or recent childbirth; (c) any condition affecting the ability to fill out the assessment, such as major neurocognitive disorders; (d) any severe neurological disorder or positive history of acute neurological injury, including an intellectual disability; and (e) the inability or refusal to provide written informed consent to participate in the study.

A semi-structured interview was administered to collect both sociodemographic and clinical characteristics. Sociodemographic characteristics included age, gender, marital and working status, education level in years, and living status, while the clinical characteristics included family history for psychiatric disorders, age of BD onset, duration of illness, presence of lifetime involuntary hospitalization, psychotic and residual symptoms, suicide attempts and non-suicidal self-injury (NSSI), lifetime SUD, and pharmacological treatment grouped as antidepressants, antipsychotics, mood stabilizers (i.e., lithium, valproate, lamotrigine, and carbamazepine), benzodiazepines, and others (i.e., gabapentin, pregabalin, and oxcarbazepine). Moreover, according to the existing literature, the presence of at least two or four medications was defined as polypharmacy simplex and complex, respectively (33).

Potential participants were provided with an in-depth explanation of the study objectives and procedures and an opportunity to ask questions. The study was designed in agreement with the guidelines from the Declaration of Helsinki (34) and was approved by the Research Ethical Committees.

### Statistical Analysis

Continuous and categorical variables were presented as means and standard deviations (SD) or frequency and percentage, respectively.

First, the sample was divided into three subgroups: (a) patients with BD and absence of lifetime SUD; (b) patients with BD and lifetime SUD (use of one illicit drug); and (c) patients with BD and polySUD (at least two concomitant illicit drugs). For the sociodemographic and clinical comparison, ANOVA with Bonferroni’s *post hoc* correction was used for continuous variables and Pearson’s chi-square test (χ^2^) for categorical variables. Subsequently, a multinomial regression analysis was used to explore the relationship between patients with BD and polySUD and each of the significant independent variables found in univariate analysis, correcting for age and gender.

The Statistical Package for Social Sciences (SPSS) for Windows 25.0 (IBM Corp., Armonk, NY, United States) was used to carry out all the mentioned statistical analyses, and the value of statistical significance was set at *p* < 0.05 (two-tailed).

## Results

### General Characteristics of the Sample

The total sample included 556 patients with a primary diagnosis of BD, of which 43.7% were men (*N* = 243). The mean (± *SD*) age was 49.17 (± 14.14) years, and 239 patients (43.0%) were single. Moreover, 198 patients (35.6%) were living alone, and 190 (34.2%) were employed. A total of 277 patients were affected by BD type I, the age of onset was 29.07 (± 13.26) years, while the duration of illness was 19.79 (± 13.10) years. Finally, about half of the patients (*N* = 225, 40.5%) were taking at least four medications.

All sociodemographic and clinical characteristics are displayed in Tables 1, 2.

**TABLE 1:** Sociodemographic characteristics of the total sample and comparison between the three subgroups.

	Total sample (*N* = 556)	BD without SUD (*N* = 376)	BD with SUD (*N* = 101)	BD with polySUD (*N* = 79)	*F*	*p*
Gender (male), *N* (%)	243 (43.7)	135 (35.9)	56 (55.4)	52 (65.8)	30.666	<0.001
Age (years), mean ± *SD*	49.17 ± 14.14	51.64 ± 13.87	47.98 ± 13.02	38.90 ± 11.99	29.718	<0.001*
Marital Status, *N* (%) Single Married Separated/divorced Widowed	239 (43.0) 180 (32.4) 108 (19.4) 29 (5.2)	137 (36.4) 140 (37.2) 70 (18.6) 29 (7.7)	47 (46.5) 30 (29.7) 24 (23.8) 0 (0)	55 (69.6) 10 (12.7) 14 (17.7) 0 (0)	44.642	<0.001
Educational level (years), mean ± *SD*	11.62 ± 3.62	11.83 ± 3.74	11.07 ± 3.21	11.33 ± 3.44	2.052	0.129
Occupational status, *N* (%)	190 (34.2)	136 (36.2)	30 (29.7)	24 (30.4)	2.069	0.355
Living with, *N* (%) Alone Family Residential facility	198 (35.6) 341 (61.3) 17 (3.1)	141 (37.5) 230 (61.2) 5 (1.3)	31 (30.7) 67 (66.3) 3 (3.0)	26 (32.9) 44 (55.7) 9 (11.4)	23.670	<0.001

*Post hoc (Bonferroni) BD without SUD > BD with SUD > BD with polySUD.

*BD, bipolar disorder; SUD, substance use disorder.*

**TABLE 2:** Clinical characteristics of the total sample and comparison between the three subgroups.

	Total sample (*N* = 556)	BD without SUD (*N* = 376)	BD with SUD (*N* = 101)	BD with polySUD (*N* = 79)	*F*	*p*
Psychiatric family history, *N* (%)	257 (46.2)	158 (42.0)	54 (53.5)	45 (57.0)	8.467	0.015
Age at onset, mean ± *SD*	29.07 ± 13.26	31.20 ± 13.88	27.29 ± 10.46	21.25 ± 9.78	20.878	<0.001*
Duration of illness (years), mean ± *SD*	19.79 ± 13.10	19.98 ± 13.41	20.99 ± 13.71	17.38 ± 10.41	1.802	0.166
Diagnosis, *N* (%) Bipolar disorder I Bipolar disorder II	277 (49.8) 279 (50.2)	187 (49.7) 189 (50.3)	45 (44.6) 56 (55.4)	45 (57.0) 34 (43.0)	2.733	0.255
Psychotic symptoms, *N* (%)	156 (28.1)	98 (26.1)	23 (22.8)	35 (44.3)	12.468	0.002
Residual symptoms, *N* (%)	92 (16.5)	51 (13.6)	16 (15.8)	25 (31.6)	15.502	<0.001
Involuntary hospitalization, *N* (%)	115 (20.7)	69 (18.4)	21 (20.8)	25 (31.6)	7.034	0.030
Lifetime suicide attempts, *N* (%)	181 (32.6)	124 (33.0)	32 (31.7)	25 (31.6)	0.095	0.953
Non-suicidal self-injuries, *N* (%)	92 (16.5)	55 (14.6)	21 (20.8)	16 (20.3)	3.107	0.212
Polypharmacy simplex, *N* (%)	101 (18.2)	75 (19.9)	18 (17.8)	8 (10.1)	4.245	0.120
Polypharmacy complex ≥ 4, *N* (%)	225 (40.5)	137 (36.4)	46 (45.5)	42 (53.2)	8.904	0.012
Antidepressants, *N* (%)	224 (40.3)	152 (40.4)	46 (45.5)	26 (32.9)	2.950	0.229
Antipsychotics, *N* (%)	423 (76.1)	291 (77.4)	70 (69.3)	62 (78.5)	3.153	0.207
Mood Stabilizers, *N* (%)	470 (84.5)	310 (82.4)	87 (86.1)	73 (92.4)	5.195	0.074
Benzodiazepines, *N* (%)	369 (66.4)	236 (62.8)	69 (68.3)	64 (81.0)	9.948	0.007
Others, *N* (%)	135 (24.3)	75 (19.9)	33 (32.7)	27 (34.2)	11.919	0.003

*Post hoc (Bonferroni) BD without SUD > BD with SUD > BD with polySUD.

*BD, bipolar disorder; SUD, substance use disorder.*

### Comparison of Sociodemographic and Clinical Parameters Between Three Groups of the Sample

Comparing the sociodemographic characteristics, patients with BD and polySUD were more frequently males (65.8% vs. 55.4% vs. 35.9%, *p* < 0.001), younger (38.90 ± 11.99 vs. 47.98 ± 13.02 vs. 51.64 ± 13.87 years, *p* < 0.001), single (69.6% vs. 46.5% vs. 36.4%, *p* < 0.001), and living in a residential facility (11.4% vs. 3.0% vs. 1.3%, *p* < 0.001) than both participants without SUD or using only one illicit substance.

Regarding the clinical characteristics, patients suffering from BD and polySUD had an earlier age onset (21.25 ± 9.78 vs. 27.29 ± 10.56 vs. 31.20 ± 13.88 years, *p* < 0.001), a higher prevalence of psychotic (44.3% vs. 22.8% vs. 26.1%, *p* = 0.002) and residual (31.6% vs. 15.8% vs. 13.6%, *p* < 0.001) symptoms, and involuntary hospitalization (31.6% vs. 20.8% vs. 18.4%, *p* < 0.001) than the other subgroups. Finally, a family history for psychiatric disorders was significantly associated with a primary diagnosis of BD and polySUD comorbid (57.0% vs. 53.5% vs. 42.0%, *p* = 0.015).

Lastly, patients suffering from BD and polySUD showed more polypharmacy complex (53.2% vs. 45.5% vs. 36.4%, *p* = 0.012) than the other subgroups and the assumption of benzodiazepines (81.0% vs. 68.3% vs. 66.4%, *p* = 0.007) and other drugs (34.2% vs. 32.7% vs. 19.9%, *p* = 0.003). All findings are reported in Tables 1, 2.

### Multinomial Regression

When the multinomial regression was performed with BD without any SUD as the reference category, male gender [OR: 2.411; 95% confidence interval (CI): 1.50–3.87; *p* < 0.001] and use of other drugs (OR: 1.819; 95% CI: 1.09–3.04; *p* = 0.023) were associated with SUD in patients affected by BD.

Furthermore, younger age (OR: 0.938; 95% CI: 0.91–0.97; *p* < 0.001), male gender (OR: 3.464; 95% CI: 1.88–6.40; *p* < 0.001), earlier age at onset (OR: 0.956; 95% CI: 0.92–0.99; *p* = 0.014), presence of psychotic (OR: 4.254; 95% CI: 1.87–6.49; *p* = 0.001), and residual (OR: 7.815; 95% CI: 3.62–16.89; *p* < 0.001) symptoms, positive family history for psychiatric disorders (OR: 1.830; 95% CI: 1.02–3.28; *p* = 0.042), and use of benzodiazepines (OR: 3.481; 95% CI: 1.61–7.53; *p* = 0.002) resulted associated with polySUD in patients with BD, compared to the reference category. All findings are displayed in Table 3.

**TABLE 3:** Multinomial logistic regression.

	BD with SUD			BD with polySUD		
	Odds Ratio	95% CI	p	Odds ratio	95% CI	p
Gender (male)	2.411	1.50–3.87	<0.001	3.464	1.88–6.40	<0.001
Age	0.990	0.97–1.01	0.345	0.938	0.91–0.97	<0.001
Marital status (single)	0.954	0.54–1.68	0.870	0.796	0.38–1.66	0.543
Living with (residential facility)	1.500	0.33–6.87	0.601	2.241	0.59–8.51	0.236
Psychiatric family history	0.058	0.99–2.50	0.058	1.830	1.02–3.28	0.042
Age at onset	0.983	0.96–1.00	0.115	0.956	0.92–0.99	0.014
Psychotic symptoms	0.719	0.41–1.28	0.261	4.254	1.87–6.49	0.001
Residual symptoms	1.699	0.86–3.36	0.127	7.815	3.62–16.89	<0.001
Involuntary hospitalization	0.976	0.52–1.80	0.939	0.955	0.47–1.94	0.898
Polypharmacy complex ≥ 4	1.288	0.77–2.16	0.338	1.570	0.83–2.98	0.168
Benzodiazepines	1.154	0.67–1.98	0.608	3.481	1.61–7.53	0.002
Others	1.819	1.09–3.04	0.023	1.450	0.63–2.78	0.158

*The reference category is: bipolar patients without substance use disorder.*

*BD, bipolar disorder; CI, confidence interval; SUD, substance use disorder.*

## Discussion

The present paper aimed to evaluate the factors associated with SUD and polySUD in a cohort of patients with BD. Evidence from epidemiological, clinical, and review studies has shown that the comorbidity between patients with a primary diagnosis of BD and SUD is frequent (23, 27, 28, 35). This has also been confirmed in our sample. Moreover, our results showed that while SUD was significantly associated only with the male gender and the category “other drugs,” several sociodemographic and clinical factors were significantly associated with polySUD compared to non-users among patients with BD. Overall, our findings underline that the presence of polySUD should be carefully considered in the pharmacological and non-pharmacological approaches to BD patients for personalized treatment.

According to the existing data literature (26, 36, 37), sociodemographic characteristics such as being male, single, and younger are associated with polySUD. These findings are quite expected, due to the increasing early use of illicit substances in young males in the current society and their well-known negative consequences on neurostructural patterns and functioning, which may impair the building of meaningful social relationships, including marital ones (38–40). However, a difference in marital status emerged only in the univariate but not in the multinomial regression analysis. Furthermore, although our findings showed that patients affected by BD and polySUD lived mainly with family members, the prevalence of patients living in residential facilities was significantly higher than in the other two subgroups. In fact, as indicated by recent guidelines, patients with BD and SUD often need to be addressed toward specific and tailored therapeutic pathways in residential facilities, especially when no familial and social support is present (41). On the one hand, this clinical indication contributes to avoid the use of illicit substances, because patients are placed in a protected context, away from the pathological environment; on the other hand, it allows to follow specific psychological and pharmacological therapeutic strategies, aimed at maintaining the mood stabilization and correcting the unhealthy lifestyles for a better social and familial functioning. Thus, the high rates of comorbidity between severe mental illness and SUD need an integrated approach in psychiatric services with specialized clinicians to encourage early assessment and intervention with active and careful monitoring (42, 43).

Regarding clinical variables, earlier age at onset, the presence of psychotic and residual symptoms, and a family history positive for psychiatric disorders were found to be significantly associated with polySUD in patients with BD. These findings are in line with other studies (35, 37, 44). Illicit substances are considered to be one of the main risk factors and triggers for bipolar illness, as defined by the new diagnostic category named “substance-induced bipolar and related disorder” in the DSM-5 (1). The use of drugs, such as cocaine, amphetamines, or cannabis, is often associated with the first manifestation of an affective episode (31, 32, 45). The earlier age at onset in people with polySUD is not surprising. A timely diagnosis of BD in individuals with SUD may represent a challenge for clinicians, as both the acute and long-term clinical effects of drug abuse and BD symptoms may overlap. Thus, the clinical picture may initially be attributed only to drug use without an accurate assessment for bipolarity, with a consequent misdiagnosis and treatment delay. The actual recognition of an underlying BD may thus be performed only during subsequent hospitalizations.

It is very important to emphasize the association between polySUD and psychotic and residual symptoms. These clinical characteristics were found to be associated with polySUD not only in the univariate but also in the multinomial regression analysis. It is well-known that psychotic and residual symptoms exert a negative impact on the functioning, quality of life, and adherence to treatment in patients with BD (46–49). The relationship between psychotic symptoms and illicit substances in BD could be bidirectional. On the one hand, as mentioned above, illicit substances may induce psychotic symptoms, especially at the onset of BD; moreover, they may determine a switch to a (hypo)manic or mixed episode and the persistence of psychotic symptoms even after the interruption of the pathological use, being potentially related to a gradual and progressive worsening of cognitive functions (50–53). On the other hand, patients suffering from BD may use drugs due to the disinhibition and exaltation typical of manic phases, or to regulate, in the short-term, the negative emotional states typical of major depressive episodes (54). The presence of residual symptoms, intra- or inter- affective episodes, are strongly associated with chronic bipolar illness course, incomplete mood stabilization and frequent affective recurrences, poor sleep quality, as well as social, occupational, and cognitive deterioration with increased subjective stigma and reduced adherence to treatment (46, 55, 56). However, data about the association between residual symptoms and SUD in patients with BD are still controversial and this relationship needs to be explored in depth (44, 57).

Family history of psychiatric disorders has been reported to be a predictor of polySUD in patients with a primary diagnosis of BD, suggesting a vulnerability for this comorbidity (35, 58, 59). Indeed, our results have shown an increase in inpatient involuntary admissions of BD patients with SUD, in line with previous studies (60, 61).

Lastly, our data revealed no difference in SUD between BD type I and II. This is in contrast with previous clinical (26, 35, 62) and epidemiological studies, reporting an increased prevalence of SUD in BD type I (20, 24). No association between polySUD and current or lifetime suicidal behaviors was found in the multinomial regression analysis, although this significant relationship has been reported in a recent meta-analysis (28). Clinicians should always monitor the bipolar illness course in patients with polySUD, focusing mainly on full clinical remission, providing more attention to psychotic symptoms, reducing involuntary admissions, and avoiding the pathological use of illicit substances to decrease suicidality, which should be addressed and evaluated with specific psychometric tools (63, 64).

Finally, with regard to pharmacological treatment, patients with BD and polySUD were more likely to be treated with a complex pharmacological approach, defined as four or more medications (33), compared to the other two subgroups. Even if some level of evidence about the efficacy of polypharmacy exists for BD with comorbid cannabis and cocaine use, unfortunately, a very low grade of evidence is available for heroin, amphetamine, and methamphetamine (41). To our knowledge, this is the first paper that analyzes this particular aspect, and to date, there is no evidence about a possible role of SUD in determining a multiple pharmacological prescriptions in patients affected by BD (33, 65). Our results may underline the necessity to develop more specific guidelines for the treatment of this peculiar subgroup of patients with BD to guide clinicians and avoid the prescription of too many medications with a poor efficacy/side effects balance.

Looking specifically at the pharmacological categories, benzodiazepines and the category of “other drugs” (i.e., gabapentin, pregabalin, oxcarbazepine) were more frequently prescribed among polySUD than the other categories. Several studies showed higher rates of use of benzodiazepines in patients with SUD than in the general population, with a risk of abuse of these drugs (66–68). On the contrary, evidence regarding factors associated with benzodiazepines prescription in patients with BD and comorbid SUD are scarce and inconclusive (69, 70). A recent review explored the use of gabapentin and pregabalin in the treatment of patients with BD, indicating a potential role of these medications in the management of SUD comorbidity (71). Similarly, oxcarbazepine has been proposed as an alternative treatment for SUD during pregnancy (72), but with scarce evidence for the treatment of SUD in comorbidity with BD (73). The increased prevalence of polypharmacy, particularly benzodiazepines and other drugs, in patients suffering from BD and polySUD should be framed as an attempt to reduce abstinence, treat residual symptoms as sleep or anxiety derived from potential subsyndromal psychotic symptoms, or recognize the need for better mood stabilization. However, these hypotheses need to be confirmed by further studies.

Despite the clinical relevance of our findings, several limitations should be discussed. First, our study has a cross-sectional design; therefore, it is not possible to make any inferences on the temporal or causal relationship between the considered variables. Second, several clinical variables (i.e., number of affective episodes, type of bipolar cycle, adherence to treatment, substance switch, and psychiatric comorbidity with personality disorders or anxiety disorders) that could affect the illness course and impact the use of specific drugs were not included in the analyses due to the high number of missing values. Third, no assessment with psychometric tools was made to investigate the potential clinical dimension such as impulsivity, hostility, and aggressiveness. Finally, our data are limited to those derived from a single research center and inpatient unit; therefore, patients with BD in a euthymic phase were not considered.

## Conclusion

Our study highlighted that several sociodemograhic and clinical characteristics may be associated with polySUD in patients with a primary diagnosis of BD. Given the significant impact of polySUD on the course and outcome of BD and the need for polypharmacy to guarantee adequate mood stabilization, further research on this topic is needed. Focusing the attention on this specific subtype of patients with BD may help implement personalized pharmacological and psychosocial therapies integrating the different professional roles.

## Data Availability Statement

The raw data supporting the conclusions of this article will be made available by the authors, without undue reservation.

## Ethics Statement

The studies involving human participants were reviewed and approved by the IRCCS Ospedale Policlinico San Martino. The patients/participants provided their written informed consent to participate in this study.

## Author Contributions

AAg and AN: statistical analyses and writing the original draft. EB, VP, and EV: data collection and revision of data literature. AAm and AC: supervision of data collection, writing the protocol, review, and editing of the original draft. LF-P: methodology, conceptualization, review, and editing of the original draft. GS, EA, and MA: scientific advisor of the project. All authors approved the final draft of the manuscript before submission.

## Conflict of Interest

The authors declare that the research was conducted in the absence of any commercial or financial relationships that could be construed as a potential conflict of interest.

## Publisher’s Note

All claims expressed in this article are solely those of the authors and do not necessarily represent those of their affiliated organizations, or those of the publisher, the editors and the reviewers. Any product that may be evaluated in this article, or claim that may be made by its manufacturer, is not guaranteed or endorsed by the publisher.
